# Mining Complex Genetic Patterns Conferring Multiple Sclerosis Risk

**DOI:** 10.3390/ijerph18052518

**Published:** 2021-03-03

**Authors:** Farren B. S. Briggs, Corriene Sept

**Affiliations:** 1 Department of Population and Quantitative Health Sciences, School of Medicine, Case Western Reserve University, 2103 Cornell Rd, Cleveland, OH 44106, USA; 2 Department of Biostatistics, Harvard T.H. Chan School of Public Health, Boston, MA 02115, USA; corriene_sept@g.harvard.edu

**Keywords:** genetic interactions, multiple sclerosis, association rule mining, epistasis

## Abstract

(1) Background: Complex genetic relationships, including gene-gene (G × G; epistasis), gene(*n*), and gene-environment (G × E) interactions, explain a substantial portion of the heritability in multiple sclerosis (MS). Machine learning and data mining methods are promising approaches for uncovering higher order genetic relationships, but their use in MS have been limited. (2) Methods: Association rule mining (ARM), a combinatorial rule-based machine learning algorithm, was applied to genetic data for non-Latinx MS cases (*n* = 207) and controls (*n* = 179). The objective was to identify patterns (rules) amongst the known MS risk variants, including HLA-DRB1*15:01 presence, HLA-A*02:01 absence, and 194 of the 200 common autosomal variants. Probabilistic measures (confidence and support) were used to mine rules. (3) Results: 114 rules met minimum requirements of 80% confidence and 5% support. The top ranking rule by confidence consisted of HLA-DRB1*15:01, *SLC30A7*-rs56678847 and *AC093277.1*-rs6880809; carriers of these variants had a significantly greater risk for MS (odds ratio = 20.2, 95% CI: 8.5, 37.5; *p* = 4 × 10^−9^). Several variants were shared across rules, the most common was *INTS8*-rs78727559, which was in 32.5% of rules. (4) Conclusions: In summary, we demonstrate evidence that specific combinations of MS risk variants disproportionately confer elevated risk by applying a robust analytical framework to a modestly sized study population.

## 1. Introduction

Multiple sclerosis (MS) is a neurodegenerative autoimmune disease of the central nervous system, and primarily affects those with European ancestry. In non-Latinx whites, the heritability of MS is estimated to be 50% (95% confidence interval [CI]: 39–61%) [1]. Additively, genetic variants explain 44.8% of the heritability for MS (*h*^2^ = 22.4%) [2,3]; thus, complex genetic (gene-gene [G × G], gene(*n*)), gene-environment (G × E), and gene-epigenome interactions, as well as intergenerational epigenetic inheritance, explain the majority of MS’ heritability (>55%) [4]. There is a modest but growing collection of G × G and G × E studies based on hypotheses derived from functional studies and/or biological knowledge that have uncovered novel risk loci and/or genetic mechanisms that begin to add context to the missing heritability in MS [5,6,7,8,9,10,11]. The principal impediments to elucidating these complex relationships is a paucity of comprehensive epidemiologic and multi-*omic* MS datasets, and the methodological and statistical challenges of detecting higher order relationships in big data [12,13].

The primary MS risk loci are the presence of HLA-DRB1*15:01 and the absence of HLA-A*02:01, which are amongst the 238 MS risk variants identified through genome-wide association studies (GWAS) [2,3]. These incompletely penetrant risk loci encompass 32 variants within the major histocompatibility complex (MHC), one X chromosome variant, five low-frequency non-MHC variants, and 200 higher-frequency non-MHC variants, and they individually confer modest risk (relative risks [RR] > 1.05 and <1.4), with the exception of HLA-DRB1*15:01 presence and HLA-A*02:01 absence (RR ≥ 1.5). MS disproportionately affects women, however, there are no sex-difference in the genetic risk for MS (with the exception of the one X chromosome variant) [14]. Bioinformatic analyses emphasize dysregulation in diverse cellular processes in adaptive and innate immunity as the principal genetic drivers of MS risk [2,15,16]. Unfortunately, specific genetic hubs have not been identified, nor have higher order genetic relationships that contribute to the liability for MS, which impedes efforts to uncover specific etiologic mechanisms. 

Over the last decade, G × G approaches have become computationally efficient while efforts aimed at discerning gene(*n*) relationships have lagged [12,13]. Several approaches rely on exhaustive two-way interaction testing following by corrections for multiple testing. This becomes infeasible for higher-order interactions due to increased computational complexity and diminished statistical power [12,13]. For example, if we were to conduct an exhaustive investigation of interactions amongst the 200 higher-frequency non-MHC MS risk variants, there would be 19,900 two-way, 1.3 × 10^6^ three-way, and 6.5 × 10^7^ four-way interactions to test. Many of the available approaches for investigating higher order interactions include a data reduction stage that reduces the search space prior investigating interactions; several of these methods have been reviewed in detailed by Niel et al. [12]. Furthermore, parametric investigations of interactions also require articulating the scale on which to investigate interactions (additive or multiplicative), as interactions are scale dependent [17].

Machine learning and data mining methods are promising approaches for uncovering higher order genetic relationships and identify genetic hubs since they are data-driven non-parametric approaches capable of navigating complex data [12,18]. However, applications of these methods in MS have been limited to investigations of G × G interactions amongst a limited set of variants or genetic heterogeneity within candidate loci [18,19,20,21]. Exhaustive searches for G × G and gene(*n*) interactions amongst the most comprehensive list of MS risk variants have not been explored, much less expansive genome-wide interaction investigations. Here, we apply association rule mining (ARM), a data mining approach that identifies frequent patterns which are used to generate association rules, to genetic data for HLA-DRB1*15:01, HLA-A*02:01, and the higher-frequency non-MHC risk variants in an exploratory effort to identify higher order relationships that contribute to MS susceptibility and add resolution to MS’ missing heritability in non-Latinx whites. 

## 2. Materials and Methods

### 2.1. Association Rule Mining

ARM is a rule-based machine learning method that relies on the a priori algorithm for efficient mining of association rules within large datasets [22,23,24]. It was originally developed for market basket analyses of patterns in retail transactions, but it has been applied to diverse relational datasets, including applications for discerning multimorbidity patterns in administrative claims data and characterizing complex genetic relationships in simulated data [25,26]. ARM requires a binary incidence matrix from which to generate itemsets: groupings of items irrespective of their order. Frequent itemsets are defined by support, which is the prevalence of the itemset in the dataset. As an itemset grows in length, support is non-increasing where P(A∩B∩C)≤P(A∩B)≤P(A) since {A}⊆{A,B}⊆{A,B,C}. Additionally, {A,B,C} cannot be a frequent itemset unless A, B, and C are frequent, as well as all other supersets since {A,B,C} is a subset of {A,B}, {A,C}, and {B,C}. These principles allow for computational efficiency by limiting the number of itemsets to be considered based on a minimum support threshold. If {A} does not meet the minimum support, then all subsets (i.e., {A,B}, {A,C}, {A,B,C}, {A,B,C,D}) will also not meet the threshold, and therefore do not need to be considered. 

Association rules are then constructed for these frequent itemsets (e.g., rule {A}→{B} for itemset {A,B}). Confidence measures the strength of an association for a rule; for example, for itemset {HLA-DRB1*:15:01 *presence*, MS} if there is a rule {HLA-DRB1*:15:01 *presence*}→{MS}, then the item {HLA-DRB1*:15:01 *presence*} provides information about the item {MS}. Confidence is $P\left(B|A\right)=\frac{P\left(A\cap B\right)}{P\left(A\right)}$, which is the probability of MS given a person has ≥1 HLA-DRB1*15:01 alleles. Unlike support, confidence is not a function of the rule’s length. For example, the rule {HLA-DRB1*:15:01 *presence,* HLA-A*02:1 *absence*} →{MS} will likely have lower support but higher confidence than the component items. However, adding noise to a rule will likely decrease support and confidence. 

Lift is another informative measure [27], which is $\frac{P(B|A)}{P\left(B\right)}=\frac{P\left(A\cap B\right)}{P\left(A\right)P\left(B\right)}$, and seeks to determine whether the left hand side (LHS) of the rule (HLA-DRB1*15:01 presence), is independent of the right hand side (RHS) of the rule (MS). If all individuals in the dataset have ≥1 HLA-DRB1*15:01 alleles (regardless of how many have MS), then $\frac{P(B|A)}{P\left(B\right)}=\frac{P\left(A\cap B\right)}{P\left(A\right)P\left(B\right)}=\frac{P\left(A\cap B\right)}{P\left(B\right)}=\frac{P\left(B\right)}{P\left(B\right)}=1$. Since P(A∩B)=P(A)P(B), these items are then independent events and this rule is not informative; this holds even if P(A)≠1 and P(B)≠1. Therefore, lift can help identify rules with limited useful information. 

### 2.2. Study Population and Genetic Data

The study population consisted of 386 unrelated non-Latinx whites (207 MS cases, 179 unaffected controls) who participated in the Accelerated Cure Project for MS. Briefly, participants were recruited from communities of 10 MS specialty clinics across the United States and eligibility criteria have been described [28]. A MS diagnosis was confirmed by a neurologist using standard diagnostic criteria at enrollment [29,30]. All participants gave informed consent and contributed biological samples from which DNA was extracted. DNA samples were genotyped using the Illumina MEGAEx BeadChip and imputed using the Michigan Imputation Server and the Haplotype Reference Consortium reference panel of ~65,000 European haplotypes. Genetic variants with an imputation quality score (r^2^) ≥ 0.8 were retained [31]. Multidimensional scaling (MDS) components were generated for a subset of independent SNPs to determine genetic outliers and cryptic relatives who were removed from the data—this too has been described [31]. 

Genetic data for HLA-DRB1*15:01 (rs3135388A), HLA-A*02:01 (rs2975033T), and 180 higher-frequency non-MHC variants were available, as were data for an additional 14 proxy variants (10 variants in linkage disequilibrium [R^2^: 0.89–1] and 4 variants reported as the discovery variants in the GWAS of MS risk [2]). Thus, a total of 194 non-MHC risk variants were investigated, which included 150 (77.3%) genic variants across 146 genes (Supplementary Table S1). Seven non-MHC variants had ≤1.3% missing observations which were further imputed using random forests single imputation (R package *missForest*). We constructed a binary incidence matrix capturing presence of a risk allele (dominant model) for all 196 risk variants; this was due to the fact that these variants were associated with MS under an additive model (therefore, having ≥1 allele conferred risk) [2] and to reduce the number of items considered (e.g., having 0, 1, or 2 HLA-DRB1*15:01 alleles would be parametrized as three items). 

### 2.3. Statistical Analyses

Rules of length 2, 3, 4, and 5 with confidence ≥80% and support ≥5% were mined using ARM (R package *arules*). Lift was not informative in this analysis for two reasons: 1. Lift = $\frac{confidence}{P\left(RHS\right)}$ and since there is only one outcome where (RHS) = MS, lift will be directly proportional to confidence; and 2. Lift can determine independence between the LHS and RHS of a rule, but since the P(MS) = 0.54 in this dataset and the confidence threshold is ≥80%, then lift for all rules will be ≥1.48, which implies the RHS (MS) and the LHS are not independent. Thus, lift will not provide additional insights for discerning strong rules from weak rules. Furthermore, we investigated itemsets that considered presence of a risk allele for a given variant; this is because the objective of this exploratory analysis was to identify higher order patterns conferring MS risk. We did not consider relationships including no copies of a risk allele for a given variant; while it would be interesting to investigate, it would significantly increase the number of possible itemsets to be considered.

Once association rules with confidence ≥80% and support ≥5% were identified, we characterized their relationships with MS using logistic regression models, to generate odds ratios (ORs) adjusting for the first three MDS dimensions to account for population substructure (STATA v13.1, StataCorp, College Station, TX, USA; command *logit*). Bootstrapping based on 5000 resamples was used to generate bias-corrected standard errors and 95% confidence intervals (CI), and normal-based *p*-values to minimize the potential impact of sampling variability (option *vce(bootstrap, 5000)*). A Bonferroni correction adjusted for multiple testing (p_corrected_ = 0.05/114 rules = 4.4 × 10^−4^). 

Given the agnostic and non-parametric nature of ARM, contextualizing the mined genetic patterns will importantly guide interpretations. Here we explored a few approaches. First, for the top ranking MS-associated rule, we parametrically characterized the relationships amongst its component variants for the presence additive (STATA command *ic*) and multiplicative interactions (STATA command *logit*). Second, in an effort to understand how rules were interconnected, network graphs were used to identify genetic variants that were items across the top 15 rules ranked by confidence. Additionally, lastly, to determine if there was any biological evidence that might provide context for observing specific subsets of rules, we explored protein-protein interactions amongst the component items using STRING v11.0, limiting interactions to those with medium confidence scores from high-throughput experiments and curated knowledge databases [32]. 

## 3. Results

The study population (*n* = 386) included 207 MS cases and 179 unrelated controls. The mean age at sample collection was 46.8 years (standard deviation [SD] = 11.0) and 46.8 years (SD = 15.7) for MS cases and controls, respectively. On average, cases reported their first MS symptom near the age 34.0 years (SD = 9.9). The female to male ratio was 3:1 in cases and 2:1 in controls. The presence of HLA-DRB1*15:01 was significantly associated with MS risk (OR = 1.89; 95% CI: 1.23, 2.91; *p* = 0.0038), as was the absence of HLA-A*02:01 (OR = 1.62; 95% CI: 1.07, 2.44; *p* = 0.023). Thus, the study population appears representative of other MS case–control studies of non-Latinx whites. 

One hundred and fourteen association rules had confidence ≥0.80 and support ≥0.05 (Supplementary Table S2). All rules had a length of four: comprised of three risk variants on the LHS and MS on the RHS. Support ranged from 0.052 to 0.104, confidence ranged from 0.80 to 0.95, and lift ranged from 1.49 to 1.78. These ranges imply that moderately common genetic combinations (support) that were much more common in MS cases than controls (confidence) were identified, and that these genetic combinations were associated with having MS (lift). The top 7 MS rules by confidence are shown in Table 1. The rule with the highest confidence (0.95) was {HLA-DRB1*15:01, *SLC30A7*-rs56678847, *AC093277.1*-rs6880809} →{MS}; this risk variant pattern existed in 21 of 386 study participants, of whom 95% were MS cases (*n* = 20) and only one was a control. By the nature of defining confidence ≥0.80, all rules would be exceptionally more common in MS cases compared to controls (Table 1; Supplementary Table S2). This is evident by their strong associations with MS (OR > 6.8 for the top 7 rules and >3.5 for all rules; *p* < 0.03). One of the rules tied for 4th rank (confidence = 0.88) included *GRB2* and *STAT3* risk variants (OR = 7.15; *p* = 0.0014; Table 1), which was reassuring since *GRB2* regulates *STAT3* [33]. 

Four rules were significant after accounting for multiple testing and HLA-DRB1*15:01 was an item in each (Table 2). The most significant rule was also the top ranking rule by confidence: {HLA-DRB1*15:01, *SLC30A7*-rs56678847, *AC093277.1*-rs6880809} →{MS}, and individuals with this genetic pattern had 20.2-fold increased odds of MS (95% CI: 8.5, 37.5; *p* = 4 × 10^−9^). Of these three risk variants, only the presence of HLA-DRB1*15:01 was significantly associated with MS in this data set, while the other two variants had associations in the expected direction (Supplementary Table S3A). This rule captured an additive interaction, evident by the stratified ORs for combinations of these risk variants and as statistically measured by an attributable proportion (Supplementary Table S3B). Ninety-four percent of the MS risk conferred by this three-way rule was due to the presence of an additive interaction (*p* < 5 × 10^−5^). On the multiplicative scale, there was also evidence for an interaction in the full parameterized model (three-way interaction term OR = 26.81; *p* = 0.02; Supplementary Table S3C).

The 114 rules were comprised of 112 unique risk variants, of which 99 were genic variants spanning 87 genes. In fact, 95.3% of the 342 possible genetic items were genic (Supplementary Table S2). Ten risk variants were items in ≥7 rules (Table 3). The risk variants that were the most frequent items were *INTS8*-rs78727559 (37 rules), *TNIP3*-rs17051321 (36 rules), HLA-DRB1*15:01 (25 rules), *SLC30A7*-rs56678847 (25 rules), and *BCL10*-rs3548693 (24 rules), suggesting these variants are probable genetic hubs in higher order genetic relationships contributing to MS risk. There were also common dyads amongst these rules, for example, 75.7% of rules including *INTS8*-rs78727559 also included *TNIP3*-rs17051321, and 80% of rules containing HLA-DRB1*15:01 also included *SLC30A7*-rs56678847 (Supplementary Table S2). Interestingly, only one rule included both MHC alleles: {HLA-DRB1*15:01, HLA-A*02:01 absence, *SLC30A7*-rs56678847} →{MS}, with support = 0.073 and confidence = 0.8 (OR = 3.99; 95% CI: 1.81, 11.97; *p* = 0.0044).

**Figure 1:**
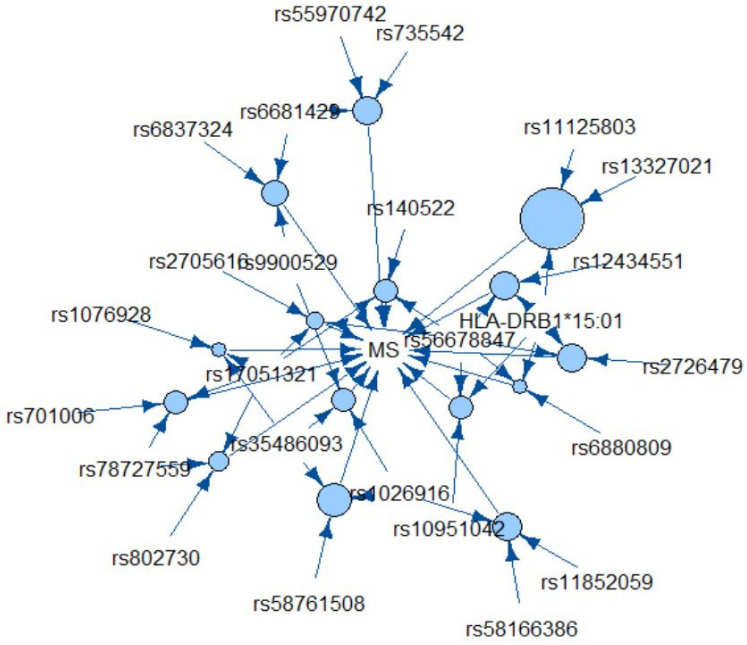
Top 15 rules by confidence. Each circle represents a rule and the arrows pointing to a circle are the LHS items in that rule. Larger circles reflect higher support.

Amongst the top 15 rules ranked by confidence, the most common items were *SLC30A7*-rs56678847 (6 rules), HLA-DRB1*15:01 (5 rules), *TNIP3*-rs17051321 (5 rules), and *BCL10*-rs3548693 (4 rules); surprisingly, the most frequent item across all rules (*INTS8*-rs78727559) was not as common amongst the top 15 rules. The overlap in the top 15 rules is visualized in a network graph shown in Figure 1.

**Figure 2:**
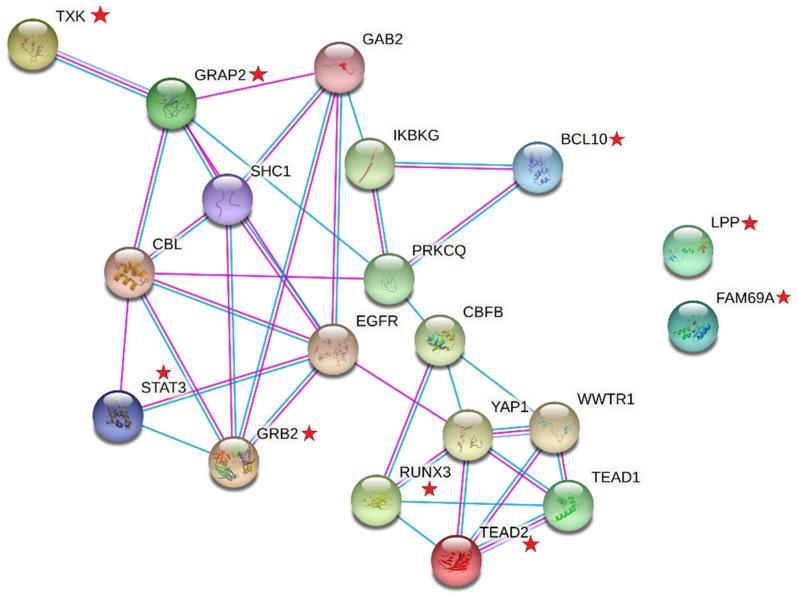
Protein-protein interactions amongst the 9 proteins (red stars) encoded by MS risk loci that were in rules with *GRB2*.

We explored protein-protein interaction networks for *GRB2* since it is a MS risk locus with substantial experimental evidence at the protein level. The objective was to explore if there was any biological evidence to complement a subset of the mined rules. *GRB2*-rs9900529 was an item in 7 rules, along with 8 other genic variants in *BCL10*, *FAM69A*, *GRAP2*, *LPP*, *RUNX3*, *STAT3, TEAD2*, and *TXK* (Supplementary Table S2). Protein-protein interactions amongst the 9 encoded proteins are shown in Figure 2, demonstrating that there are biological interconnections amongst the proteins encoded by the genes represented in these mined patterns.

## 4. Discussion

The majority of MS’ heritable component has yet to be discovered. While large-scale and collaborative GWAS and targeted functional studies will importantly continue to uncover risk loci, these variants will additively explain only a portion of MS’ heritability (*h*^2^ = 22.4%) [2,3,4]. Complex genetic/epigenetic relationships (i.e., G × E and gene(*n*) interactions), including those amongst the GWAS-identified MS risk loci, will explain the majority of MS’ heritability. A handful of studies with limited scope have begun to disentangle these complex features in MS’ heritability, but none have explored higher order relationships amongst MHC and non-MHC risk variants [5,6,7,8,9,10,11]. Here, we present an application of a combinatorial, data mining algorithm as a computationally efficient method for delineating higher order relationships contributing to MS’ liability. Using ARM and genetic data for 196 risk variants (2 MHC and 194 non-MHC variants) in 386 subjects, we successfully mined 114 genetic patterns. These patterns were three-way combinations of MS risk loci, and the mined patterns were common in the study population (frequency: 5.2% to 12.7%) but substantially more so in MS cases than controls (ORs ≥ 3.6; *p* < 0.03). 

After imposing a multiple testing correction, there were four genetic patterns that were significantly associated with MS risk (ORs: 4.9 to 20.2; *p*: 4.1 × 10^−4^ to 4.4 × 10^−9^). HLA-DRB1*15:01 was a shared attribute across these genetic rules and *SLC30A7*-rs56678847 was common in two of the four. In fact, HLA-DRB1*15:01 and *SLC30A7*-rs56678847 existed in 21.9% of all 114 rules, and jointly occurred in 17.5% of rules. It was not unexpected that HLA-DRB1*15:01 was a common feature since it is the predominant MS risk factor. What was interesting was that the absence of HLA-A*02:01 was only in one of these HLA-DRB1*15:01 rules, and that *SLC30A7* was a part of 80% of them, suggesting a possible genetic dyad hub. The rule with the strongest association (OR = 20.2; *p* = 4.4 × 10^−9^), which captured interactions on both the additive and multiplicative scales, included this dyad and rs6880809 located in *AC093277.1*, a long non-coding RNA associated with several autoimmune diseases but whose function is unknown (https://www.genecards.org/cgi-bin/carddisp.pl?gene=ENSG00000283286 (accessed on 28 January 2021)). In a post hoc analysis, the HLA-DRB1*15:01-*SLC30A7* dyad occurred in 9.8% of the study population and had support = 0.075, confidence = 0.76, and OR = 3.21 (95% CI: 1.47, 7.00; *p* = 0.0034). *SLC30A7* encodes the ubiquitously expressed zinc transporter 7 (ZNT7), which facilitates zinc transport into the Golgi apparatus and regulates cellular zinc homeostasis [34]. Zinc has been implicated in the pathogenesis of MS, including polarization of macrophages [35,36,37]. However, most relevant to the HLA-DRB1*15:01-*SLC30A7* dyad is evidence that zinc facilitates MHC Class II dimerization which impacts antigen binding and presentation [38] and that *Slc30a7* is differentially regulated in CD4+ T cells in a MS mouse model [39]. 

*INTS8*-rs78727559 was the most common risk variant across the 114 rules (32.5%), followed by *TNIP3*-rs17051321 (31.6%), and jointly occurred in 24.6% of all rules. In a post hoc analysis, this dyad was present in 7.5% of the study population and had support = 0.06, confidence = 0.79, and OR = 3.62 (95% CI = 1.42, 9.22; *p* = 0.007). As a dyad, or dyads in 28 of 114 mined triads, this is an interesting but less obvious pairing that merits further investigation. *INTS8* is highly expressed in the brain, plays a significant rule in neuronal and brain development, and mutations are associated with rare recessive neurodevelopmental syndromes [40]. Additionally, *intS8* knockdown suppresses intermediate neural progenitor dedifferentiation in *Drosophila* [41]. *TNIP3* is a TNFAIP3 interacting protein that is highly expressed in lymph nodes, thymus, and expressed at lower levels in the brain and other tissues. TNIP3 binds to TNFAIP3 to inhibit NF-κB activation, but TNIP3 can also inhibit NF-κB in response to lipopolysaccharides (LPS; potent stimulators of innate immunity) [42]. This latter fact may relate to LPS-induced and NF-κB-controlled microglial neuroinflammation in MS mouse models [43,44]; though we are speculating. Thus, the dyad of *INTS8* and *TNIP3* might reflect a nexus between neuroinflammation and diminished neuronal repair.

Several of the other genetic rules merit closer examination, i.e., those with *GRB2* and *STAT3*, given GRB2 regulates STAT3 [33]. An exploratory analysis of MS risk loci within rules including *GRB2* suggests that these rules might reflect both statistical and biological relationships—however, functional analyses are warranted (Figure 2). Thus, ARM represents a powerful and efficient algorithm capable of extracting meaningful relationships that might illustrate novel or key genetic mechanisms underlying MS susceptibility. By using specific thresholds for support and confidence, we conserved power; for example, an exhaustive search of two to four-way interactions would have resulted in 6.7 × 10^7^ interactions to be tested. Other strengths of this exploratory investigation are the opportunity to generate complex genetic hypotheses in MS, utilizing a representative non-Latinx white MS case–control study population, and the inclusion of parametric bootstrapped models to characterize mined combinatorial relationships. The primary limitation is the sample size and therefore we were restricted to mining common rules (support ≥ 0.05); thus, it is possible undetected rare genetic patterns with stronger associations may exist. A second limitation is the absence of an independent dataset to confirm that observed associations; however, bootstrapping was used to minimize the potential impact of sampling variability. Additionally, lastly, while we investigated MS-associated risk loci, in the absence of fine-mapping analyses, it is not known if these variants are the causal MS variants. The MS risk variants in Table 2 and Table 3 are either intronic or intergenic and in linkage disequilibrium with >300 variants (intronic, intergenic, and 3′/5′ UTR variants; Supplementary Table S4). Since the causal variants are currently not known, and that many of these variants are expression quantitative trait loci (eQTL) (Supplementary Table S4; detailed eQTL analyses were reported by the International MS Genetics Consortium [2]), our efforts to biologically interpret the enriched genetic patterns is challenging but merits closer bioinformatic scrutiny.

Future research should confirm the associations for these genetic patterns in an independent study population (i.e., testing if having ≥1 risk alleles at HLA-DRB1*15:01, *SLC30A7*-rs56678847, and *AC093277.1*-rs6880809 is associated with MS risk with a similar magnitude and confidence). There are opportunities to expand on the current findings, including analyses of a binary incidence matrix that captures risk allele counts per variant, mining rules for a specific variant using more liberal confidence and support thresholds (i.e., requiring a specific risk variant to be present on the LHS), and extending analyses to GWAS investigations. To the best of our knowledge, ARM has not been used in the context of a GWAS, however scalable ARM algorithms capable of analyzing GWAS data are currently in development [45], as well as frameworks that combine ARM with other deep learning or machine learning algorithms to interrogate GWAS data [46,47].

## 5. Conclusions

ARM discerned novel higher order relationships amongst MS risk variants. These complex genetic patterns had strong associations with MS; i.e., HLA-DRB1*15:01-*SLC30A7*-rs56678847-*AC093277.1*-rs6880809 conferred 20.2-fold (95% CI: 8.5, 37.5; *p* = 4 × 10^−9^) increased MS risk. In overview, we presented an analytical framework for discern features in the missing heritability of MS that is independent of parametric model assumptions and computationally efficient. Furthermore, we highlight possible genetic hubs that might be involved in several pathological mechanisms in MS. These findings may also inform genetic risk prediction efforts, particularly given the strong and robust associations observed in this modestly sized study population. 

## Figures and Tables

**Table 1:** Top seven rules by confidence.

Genetic Rule	Support	Confidence	Odds Ratio (95% CI)	*p* Value ^1^	Frequency in Controls (*n* = 179)	Frequency in MS Cases (*n* = 207)	Genes
HLA-DRB1*15:0 rs56678847 rs6880809	0.052	0.95	20.24 (8.48, 37.46)	**4.4 × 10^−9^**	0.6%	9.7%	*HLA-DRB1* *SLC30A7* *AC093277.1*
HLA-DRB1*15:01 rs56678847 rs12434551	0.065	0.89	8.50 (3.20, 31.65)	**4.1 × 10^−4^**	1.7%	12.1%	*HLA-DRB1* *SLC30A7* *ZFP36L1*
rs6681429 rs6837324 rs9900529	0.062	0.89	7.71 (2.64, 28.12)	9.6 × 10^−4^	1.7%	11.6%	*FAM69A* *TXK* *GRB2*
HLA-DRB1*15:01 rs56678847 rs10951042	0.060	0.88	7.64 (2.85, 28.05)	6.9 × 10^−4^	1.7%	11.1%	*HLA-DRB1* *SLC30A7* *LOC105375130*
rs35486093 rs1026916 rs9900529	0.060	0.88	7.15 (2.60, 26.95)	0.0014	1.7%	11.1%	*BCL10* *STAT3* *GRB2*
rs56678847 rs17051321 rs140522	0.060	0.88	7.61 (2.62, 28.49)	0.0014	1.7%	11.1%	*SLC30A7* *TNIP3* *ODF3B*
rs56678847 rs2705616 rs17051321	0.054	0.88	6.88 (2.38, 27.08)	0.0026	1.7%	10.1%	*SLC30A7* *AFF1* *TNIP3*

^1^ Bolded *p* values met the Bonferroni-corrected significance threshold.

**Table 2:** Top four rules by logistic regression bootstrapped *p*-value.

Genetic Rule	Support	Confidence	Odds Ratio (95% CI)	*p* Value ^1^	Frequency in Controls (*n* = 179)	Frequency in MS Cases (*n* = 207)	Genes
HLA-DRB1*15:01 rs56678847 rs6880809	0.052	0.95	20.24 (8.48, 37.46)	**4.4 × 10^−9^**	0.6%	9.7%	*HLA-DRB1* *SLC30A7* *AC093277.1*
HLA-DRB1*15:01 rs11125803 rs13327021	0.096	0.86	6.76 (3.13, 20.88)	**1.1 × 10^−4^**	3.4%	17.9%	*HLA-DRB1* *ADCY3* -
HLA-DRB1*15:01 rs13327021 rs735542	0.104	0.82	4.85 (2.36, 11.97)	**1.7 × 10^−4^**	5.0%	19.3%	*HLA-DRB1* - *LOC105375752*
HLA-DRB1*15:01 rs56678847 rs12434551	0.065	0.89	8.50 (3.20, 31.65)	**4.1 × 10^−4^**	1.7%	12.1%	*HLA-DRB1* *SLC30A7* *ZFP36L1*

^1^ Bolded *p* values met the Bonferroni-corrected significance threshold.

**Table 3:** The 10 most frequent risk variants in the 114 rules.

SNP	Chr	Base Pair (hg19)	Gene	Count (%)	Count in Top 15 Rules Ranked by Confidence (%)
rs78727559	8	95,851,818	*INTS8*	37 (32.5%)	1 (6.7%)
rs17051321	4	122,119,449	*TNIP3*	36 (31.6%)	5 (33.3%)
HLA-DRB1*15:01	6	32,489,683	*HLA-DRB1*	25 (21.9%)	5 (33.3%)
rs56678847	1	101,422,963	*SLC30A7*	25 (21.9%)	6 (40.0%)
rs35486093	1	85,729,820	*BCL10*	24 (21.1%)	4 (26.7%)
rs1026916	17	40,529,835	*STAT3*	12 (10.5%)	3 (2.0%)
rs11852059	14	52,306,091	*GNG2*	11 (9.6%)	1 (6.7%)
rs735542	8	128,175,696	*LOC105375752*	11 (9.6%)	1 (6.7%)
rs58166386	19	16,559,421	*EPS15L1*	7 (6.1%)	1 (6.7%)
rs9900529	17	73,335,776	*GRB2*	7 (6.1%)	2 (13.3%)

## Data Availability

These data are available from Accelerated Cure Project for MS to qualified investigators, once institutional agreements have been reached.

## Supplementary Materials

The following are available online at https://www.mdpi.com/1660-4601/18/5/2518/s1, Table S1: MS risk variants included in the analysis. Table S2: Rules mined with confidence ≥0.8 and support ≥0.05. Table S3: Testing for additive and multiplicative interactions for the top ranking rule. Table S4: Haploreg annotation of MS risk variants reported in Table 2 and Table 3.
